# Optimized barley phytase gene expression by focused FIND-IT screening for mutations in *cis*-acting regulatory elements

**DOI:** 10.3389/fpls.2024.1372049

**Published:** 2024-03-01

**Authors:** Claus Krogh Madsen, Charles Alistair Brearley, Jesper Harholt, Henrik Brinch-Pedersen

**Affiliations:** ^1^ Department of Agroecology, Aarhus University, Slagelse, Denmark; ^2^ School of Biological Sciences, University of East Anglia, Norwich Research Park, Norwich, United Kingdom; ^3^ Carlsberg Research Laboratory, J. C. Jacobsens Gade 4, Copenhagen, Denmark

**Keywords:** CRE, promoter, phytase, barley, phytate, FIND-IT, mutagenesis, Digital PCR

## Abstract

**Introduction:**

Induced modification of plant gene expression is of both fundamental and applied importance. Cis-acting regulatory elements (CREs) are major determinants of the spatiotemporal strength of gene expression. Yet, there are few examples where induced genetic variation in predetermined CREs has been exploited to improve or investigate crop plants.

**Methods:**

The digital PCR based FIND-IT technology was applied to discover barley mutants with CRE variants in the promoter of the nutritional important barley grain phytase (*PAPhy_a*) gene.

**Results and discussion:**

Mutants with higher or lower gene expression and ultimately higher or lower mature grain phytase activity (MGPA), respectively, were discovered. Field trials and inositol phosphate profiling during germination showed that *PAPhy_a* does not influence agronomic performance under the trial conditions but it does shorten the lag time of phosphate mobilization during germination. Higher endogenous MGPA is an improvement of grain quality for feed use as it improves the phosphate bioavailability for monogastric animals. Moreover, as the targeted CRE motifs of the *PAPhy_a* promoter are shared with a range of seed expressed genes like key cereal and legume storage genes, the current results demonstrates a concept for modulating individual gene expression levels of a range of seed genes.

## 1 Introduction

The current work aims to explore and demonstrate the possibility of optimizing the expression level of a single gene conferring an attractive trait by using strong molecular screening techniques to identify induced mutations with regulatory impact.

The spatiotemporal expression pattern of genes is governed by the interaction between *cis*-acting regulatory elements (CREs) and *trans*-acting transcription factors. Variations in gene expression may originate from genetic variation in CREs as well as transcription factors (Marand et al., 2023). Generally genetic variation in CREs affect the downstream gene (which may in turn regulate others) whereas *trans*-acting factors generally directly control multiple genes. Differences in gene expression levels and spatiotemporal expression pattern caused by variation in *cis*-acting regulatory elements (CREs) and transcription factors have contributed to the domestication, diversification and continued improvement of crop plants (Meyer and Purugganan, 2013). From the point of view of crop improvement, targeting CREs is a precision approach focusing on one gene whereas targeting transcription factors may modify entire regulatory networks. Both strategies have their place, but the scope of the current work is to modify single gene expression and therefore focus on CREs. As an approach to target single genes, mutations in CREs are often considered less pleiotropic than mutations in coding regions because they allow for changes that are more restricted in time and space. For instance, a CRE mutation can silence a gene in a specific cell type while the gene is expressed normally in other cell types (Wittkopp and Kalay, 2012). Mutations causing amino acid changes or perturbation of the open reading frame (e.g. premature stop codons) are frequently detrimental to protein function and will be so in all cells where the gene is expressed. On the other hand, gain, loss and modification of CREs allow for a modular type of evolution which exploit preexisting proteins in new contexts. Target CREs for breeding or research purposes may be identified from the gene specific literature or bioinformatic tools that search for known sequence motifs. Although CREs of a specific gene may be classified as enhancers, repressors etc. (for a recent review consult (Marand et al., 2023)) it is currently not possible to accurately predict the outcome of CRE mutations. Nevertheless, known or predicted CREs are sites where a mutation has a high probability of affecting gene expression. It has been proposed that site directed nuclease based genome editing technologies such as the CRISPR-Cas system could be used to generate allelic series of candidate CREs in order to create incremental variations of a target trait (Scheben and Edwards, 2018). The current work show that this can also be achieved by conventional induced mutagenesis when strong molecular screening technologies are used to identify the desired mutants.

Phytate (InsP_6_) is the major storage compound for phosphorus in seeds. Generally, around 70% of seed phosphorous is phytate bound. Seed phytate is often found as solid mixed salts known as phytin (Raboy, 1997). In cereals, phytin forms globoids mainly in the aleurone layer (small grain cereals) or embryo (maize) (O'Dell et al., 1972). In addition to phosphorus, the globoids are the main location of seed potassium and magnesium (Bethke, 1998). Calcium, iron and zinc are also present in high amounts (Bohn et al., 2007). The phytin bound nutrients are mobilized during germination by a combination of organelle acidification and the action of phytases which dephosphorylate the InsP_6_ in a sequential manner creating lower inositol phosphates (Madsen and Brinch-Pedersen, 2020). Phytate is a compound of global significance in two main areas. First, it is a major sink of phosphate in biological systems and thus impact the management of this limited resource. Specifically the very poor natural ability of monogastrics to digest phytate can lead to excessive phosphorus application to fields and ultimately the surrounding environment in areas with intensive livestock farming (Brinch-Pedersen et al., 2002). Secondly, the chelating properties of phytate negatively influence the absorption of certain cationic micronutrients by humans. This has been most clearly demonstrated in the case of zinc (Hambidge et al., 2008). Thus management of phytate e.g. by the application of phytases in the relevant contexts is important to the sustainable development goals 3, 6, 12 and 14 (United Nations, 2015).

Phytases are generally synthesized *de novo* during germination in response to gibberellin. A notable exception to this rule is the Triticeae cereals which include wheat and barley. They express one phytase, *PAPhy_a*, in high amounts during grain filling and store it in close proximity to the phytin. The stored phytase is present in the mature grain where it confers a quantifiable quality “mature grain phytase activity” (MGPA). A second phytase, the highly homologous paralog *PAPhy_b* (86% identity at the protein level in barley), is mainly expressed during germination thus following the pattern seen in plants lacking MGPA formed during seed development (Dionisio et al., 2011; Brinch-Pedersen et al., 2014). The dual phytase system has evolved after a duplication of the ancestral single *PAPhy* gene and the major consequence is the acquisition by *PAPhy_a* of a grain filling active promoter (Madsen et al., 2013).

The *PAPhy_a* and -*b* genes are preserved in diverse Triticeae species and in the homeologous loci of the polyploids, residing on chromosomes 5 and 3 respectively (Madsen et al., 2013, Madsen et al., 2017). Further, to our knowledge there are no reports of varieties of any Triticeae cereal that lack the high MGPA indicating an active *PAPhy_a* gene. This strongly suggest that *PAPhy_a* confers a significant adaptive advantage. Nevertheless, non-Triticeae cereals and plants in general germinate and complete their lifecycle with MGPA levels two orders of magnitude lower than the Triticeae (Eeckhout and De Paepe, 1994). Thus, it remains unknown which benefit *PAPhy_a* provides to the plant. However, it has been shown that PAPhy_a is a very potent and stable enzyme and that the associated high MGPA provides nutritional benefits to monogastric animals fed with the grains (Scholey et al., 2017; Holme et al., 2017a). These animals lack sufficient endogenous or gut microbial phytase activity to hydrolyze feed phytate. Consequently, the majority of feed phosphorus - as well as important cations chelated by the phytate in the digestive tract – is excreted (Brinch-Pedersen et al., 2002). Since the, 1990’s it has become common practice in many developed nations to supplement pig- and poultry feed with 500-1000 FTU/Kg of microbial phytase, where one FTU is the amount of enzyme that liberates 1 μmole of inorganic phosphorus per minute from sodium phytate at pH 5.5 and 37°C. Triticeae grains provides activities in the same range and their efficiency has been proven in feeding trials (Pointillart et al., 1987; Scholey et al., 2017). The prospect of improving MGPA in cultivars and developing feed processing technologies that preserve their activity has however received comparatively less attention.

The most striking feature of the *PAPhy_a* promoter is the composite CRE (Madsen and Brinch-Pedersen, 2019). The individual CRE motives in this element are associated with cereal and legume storage proteins where they occur in different sequence contexts. This suggest that the composite CRE is involved in the grain filling specific expression and the element is an obvious target for creating *cis*-regulatory variants (Madsen et al., 2013). Deletion of the composite CRE, except for the first and last base pair, by CRISPR-Cas9 technology reduced the MGPA of barley by 39% (Holme et al., 2017b). Greater reductions were seen by TALEN- and CRISPR-Cas9 generated deletions between the composite CRE and the TATA-boxes, whereas deletion including the translational start reduced the MGPA to less than 10% of the isogenic control (Holme et al., 2017b). In another study, 1, 2, 3, 4, and 6 extra *HvPAPhy_a* genomic copies in barley demonstrated a perfect positive linear correlation with the level of MGPA (Holme et al., 2020). These studies unequivocally confirmed that *PAPhy_a* is almost exclusively responsible for barley MGPA and, further showed that the promoter region between the composite CARE and the TATA-boxes is more important than sequence analysis has suggested. None of the deletions increased MGPA, but the strong dose effect from multiple gene copies prove that the expression is not restrained by negative feedback mechanisms.

While regulatory mutations are common in crop evolution, there is a paucity of examples where this has been exploited for targeted breeding efforts. It has been suggested that genome editing technology such as the CRISPR/Cas9 system is on the verge of changing this (Swinnen et al., 2016). While we support this view, it should be added that technologies to screen chemically or otherwise induced mutant populations is also progressing. Recently, FIND-IT was reported as a novel approach to screen large mutant populations (Knudsen et al., 2022). FIND-IT combines a hierarchal pooling strategy with a digital PCR dual hydrolysis probe assay (Pekin et al., 2011).

Here we apply FIND-IT to find novel *PAPhy_a* mutants in barley and we show that MGPA can be increased as well as decreased by chemically induced mutations targeting CREs. Further, we examine the implications of modified preformed phytase activity on the agronomic performance and on inositol phosphate metabolism during germination.

## 2 Materials and methods

### 2.1 Mutant discovery

Barley (*Hordeum vulgare* L.) cv. RGT Planet was mutagenized with 5mM sodium azide and screened by FIND-IT as described by (Knudsen et al., 2022). Up to, 480000 M_3_ lines were evaluated with primer/probe sets focusing on the composite CRE and secondly on the region between the composite CRE and the first TATA-box. Based on results of Knudsen et al. (2022) and an approximately linear correlation between mutagen and mutation rate in the concentration range [as observed by Prina and Favret (1983)], the population was estimated to have 7,4 variants per 10^6^ base pairs. Homozygous plants were selected in the M_4_ or M_5_ generation, with preference for plants most closely resembling the visual phenotype of the parent lines. Selected offspring were multiplied in the greenhouse to achieve enough material for the field trials. The promoter and coding sequence of the *HvPAPhy_a* mutants were amplified and sequenced using primers 1-4 (**Table 1**).

**Table 1:** Primers used for mutant validation (1-4) and qPCR (5-10).

Primer no.	Name	Sequence (5’→3’)	Primer efficiency	Use
1	HvPAPhy_a Pro Fw	CTCTAACCATCCAACCG	N/A	Amplification for sequencing
2	HvPAPhy_a Pro Rv	ATATCAGAGTCCGGCGAA	N/A	
3	HvPAPhy_a CDS Fw	TGCGGGTCCAAGATGAGT	N/A	
4	HvPAPhy_a CDS Rv	TGCACGGGGCGATATACT	N/A	
5	qHvPAPhy_a Fw3	TTCGGCCATGGCATCCTC	98.9%	Expression analysis
6	qHvPAPhy_a Rv2	ACAGAGCGTGCGTCTCGT		
7	qHvPAPhy_b Fw	TTCGGCCACGGCATTCTT	96.1%	
8	qHvPAPhy_b Rv2	ACAGAGCGTGCGTCTCAT		
9	qHvActin Fw	CCTCAGTTGAGAAGAGCTACG	97.6%	
10	qHvActin Rv	TCTGCGCCAATCGTGATC		

### 2.2 Field trials

Field trials were conducted in Middelfart - Denmark (2019 - N: 55.480814°, E: 9.833227°). Seeds of all mutants (LIM5, LIM7, LIM13) and their corresponding control lines (RGT Planet) treated with Redigo Pro FS 170 (Bayer Crop Science) (0.5 ml per kg) were sown 10th April, 2019 in a fine sandy soil with a sowing density of 3.6 times thousand kernel weight of each line per 10.5 m^2^ yield plot. The field received 300 kg NPK (10-9-18) and 275 kg NS (27-4) per hectare for standard fertilization levels suitable for malting barley production. The field was treated with fungicides according to standard agricultural procedures. All plots were harvested 7th August by a plot combiner of GPS controlled plot size of 8 m^2^.

### 2.3 Analyses of yield parameters

Prior to grain analysis, the harvested material was sorted above 2.0 mm in order to remove impurities and straw/leaf residues. The yield of each plot was recorded and thousand kernel weight of mature dry grains (sample size of approx. 500 kernels) were determined using a digital seed analyzer MARVIN (GTA 364 Sensorik GmbH). Statistical analyses were performed using XL-STAT (Addinsoft, France).

### 2.4 Expression analysis

Developing spikes of greenhouse grown plants were collected and immediately processed for RNA purification. Two to five seeds were collected from one row. The awns were removed and the seeds were immediately ground in a ceramic mortar with the aid of liquid nitrogen. RNA purification proceeded as described by (Chomczynski and Sacchi, 2006) with a modification to remove co-purified carbohydrates; after the final precipitation, RNA was selectively re-dissolved in formamide at 60 °C, precipitated again with isopropanol and finally redissolved in water. The corresponding seeds from the other row were used to score the seeds on the Zadoks developmental scale (Zadoks et al., 1974). RNA concentration was measured on a NanoDrop spectrophotometer and integrity was confirmed by agarose gel electrophoresis. Samples of germinating seeds were prepared by incubating seeds from the field trial at 42°C for 72 hours to break seed dormancy. Each line was represented by seeds from three different field plots. The seeds were then surface sterilized for ten minutes in 0.5% sodium hypochlorite and washed twice with sterile water. The sterilized seeds were laid out on filter paper wetted with 7 mL sterile water in petri dishes (9 cm diameter). Only healthy and typical looking seeds were used, and all the seeds were oriented with the crease down and positioned with regular spacing. The plates were placed in clear plastic bags to reduce evaporation and placed in a plant incubator at 23°C and a 16/8 day/night cycle. Two seeds were collected from each biological repetition at each time-point, 24 hours (1 day after imbibition (dai)) and 72 hours (3 dai) respectively. The seeds were immediately processed for RNA purification as described above.

Reverse transcription was performed with Superscript IV reverse transcriptase and a 20dT primer essentially according to the manufacturer’s instructions. The synthesis was allowed to proceed at 50°C for 5 minutes followed by 15 minutes at 55°C. 1.1 µg of total RNA was used provided that the RNA concentration was > 100 ng/µL. The lowest RNA amount was 0.75 µg.

Quantitative real time PCR was performed in 12 µL 1x Power SYBR Green PCR Master Mix on a ViiA7 real time PCR instrument (both Applied Biosystems) using primers 5-10 (**Table 1**). All combinations of barley lines and developmental stage were analyzed in at least three biological repetitions, each measured in technical triplicate. The qHvActin Fw/Rv reference gene primers were reported and validated previously (Kaczmarczyk et al., 2012). The qHvPAPhy_a Fw/Rv and qHvPAPhy_b Fw/Rv primer sets were developed for the current study. Both span the last intron of the respective genes.

### 2.5 Germination experiment

Fifteen grams of seeds from each field trial plot were incubated at 42°C for 72 hours to break seed dormancy. The seeds were then surface sterilized for ten minutes in 0.5% sodium hypochlorite and washed twice with sterile water. The sterilized seeds were laid out on filter paper wetted with 10 mL sterile water in 12 by 12cm square petri dishes (42 seeds per plate). Only healthy and typical looking seeds were used, and all the seeds were oriented with the crease down and positioned with regular spacing. The plates were placed in clear plastic bags to reduce evaporation and placed in a plant incubator at 23°C and a 16/8 day/night cycle. Twenty representative seeds were collected each day from all the replications. The samples were immediately frozen in liquid nitrogen and kept at -80°C until all samples had been collected. The material was lyophilized for eight days.

*Phytase assay*. Mature and lyophilized germinating grains were assayed for phytase activity as described by (Engelen et al., 1994) with modifications. The material was ground to a fine powder on an IKA tube-mill. Protein was extracted from flour using 10 ml of 25 mM sodium acetate buffer (pH 5.5) with 0.1 M calcium chloride per gram and shaking for 60 minutes. After centrifugation, 30 μl was diluted to 800 μl with extraction buffer containing sodium phytate to a final concentration of 5 mM. Phytate hydrolysis proceeded for 60 minutes at 37°C. The reaction was stopped with 800 μl of freshly prepared 25 g/l ammonium heptamolybdate with 0.25% ammonia: 6.9 g/l ammonium vanadate with 1% nitric acid: 65% nitric acid (15:15:10). The mixture was centrifuged for 5 minutes at 10000×g before absorbance was measured at 415 nm. Blanks were included for each sample by substituting the substrate with extraction buffer and omitting the incubation at 37°C. Using blanks for each sample allowed the subtraction of background absorbance from free phosphate. The phytase activity was calculated according to a standard curve. All samples were measured in technical triplicates. The phytate substrate was prepared and tested as described by (Madsen et al., 2019).

### 2.6 Quantification of inositol phosphates

Inositol phosphates were extracted from the same flour samples as used for the phytase assay. They were separated by anion exchange HPLC on a 3 mm×250 mm CarboPac PA200 column (Dionex, Sunnyvale, CA) fitted with a 3 mm×50 mm guard column of the same material. The column was eluted with a gradient of methanesulfonic acid delivered at a flow rate of 0.4 mL min^−1^ by mixing of solvents from reservoirs containing water (A) and 0.6M methanesulfonic acid (Acros Organics) (B) according to the schedule: time (min), %B; 0,0; 25,100; 38,100; 39,0; 49,0. The column eluate was mixed in a mixing Tee with a solution of 2% w/v ferric nitrate (nonahydrate) in 0.1% v/v perchloric acid delivered at a flow rate of 0.2 mL min^−1^. The combined flow was passed through a 4 m×0.25 mm i.d. knitted reaction coil (Biotech AB, Sweden) and inositol phosphate peaks were monitored by UV absorbance at 290 nm (Phillippy and Bland, 1988). Peak areas were integrated with ChromNav (Jasco) software and compared to that of standards of Na_12_InsP_6_ (Merck Millipore – Calbiochem Cat: 407125-25 MG Lot: 2663470). The Jasco LC-4000 HPLC system comprised: an AS-40140 autosampler, PU-4085i and PU4080i pumps, a UV-4070 UV-visible detector and a CO-4061 column oven. An acid-hydrolysate of phytate was obtained by refluxing 3 g sodium phytate (Sigma P8810) in 100 mL of 1M HCl for 24 h. The resulting solution was lyophilized to near dryness, made up to 100 mL with water, lyophilized again and made to a final volume of 100 mL with water. For use as an HPLC standard, the hydrolysate was diluted 20-fold with water and 20 μL injected onto HPLC. For reproduction of HPLC profiles, data was exported from ChromNav as an ASCII file and redrawn in GraFit v.7 (Leatherbarrow, 2010).

### 2.7 Statistical analysis

The phytase activity during germination was evaluated by comparing the measured activity at a given time point to the mean of RGT Planet’s measured activities at the same time. These relative means were then analyzed by ANOVA multiple comparison (Tukey test), using an Excel plugin (XL-STAT, Addinsoft, France).

As described above, phytate content in germination grains was analyzed by ANOVA multiple comparisons. The rate of phytate degradation was calculated by comparing measured phytate to mean phytate content of the same genotype at day 0 and then analyzed by ANOVA multiple comparisons. Outliers were called by Grubbs test (Grubbs, 1969).

## 3 Results

### 3.1 Identified mutants

**Figure 1:**
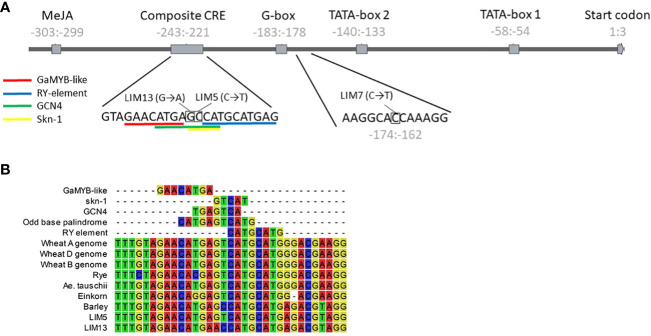
**(A)** Structure of the *HvPAPhy_a* promoter and positions of the LIM5, 7 and 13 mutations. The position of the promoter elements relative to the start of the coding sequence is given in gray numbers. The sequence context of the mutations is shown below the graphic representation. **(B)** Natural and induced variation in the composite CRE. Alignment of the sequence from selected Triticeae and the LIM5 and LIM13 mutants. Individual CRE sequences are included for reference.

Screening of the mutant library led to the discovery of three *PAPhy_a* promoter mutants, LIM5, LIM7 and LIM13. The mutants were cross-checked with conventional PCR and sequencing to confirm the nature of the mutation and verify that no other mutations were present in the core promotor or CDS region. At this point the mutants were of the M4 generation. The region amplified for sequencing by primers 1-4 (**Table 1**) comprised the CDS, 794 bp upstream and 58 bp downstream. LIM5 and LIM13 harbor mutations in the known composite CRE, whereas LIM7 has a mutation closer to the translational start codon at a position without obvious CRE, (**Figure 1**).

### 3.2 MGPA and agronomic performance of the mutants

The MGPA of homozygous M_4_ or M_5_ mutants and parent RGT Planet cultivar was preliminarily assessed using the small number of greenhouse grown plants available. The LIM5 mutant showed an average increase of 26% and LIM13 showed a decrease of 22% compared to the parent. LIM7 showed a reduction of 88%. Grains from the field trials confirmed these trends (**Table 2**). The field trials were also used for evaluating the agronomical performance of the LIM mutants. Both plot yields and thousand grain weight (TGW) of LIM5 and LIM7 were unchanged compared to RGT Planet, for LIM13 decreases in both TGW and yield were observed (Tukey multiple comparison ANOVA, n=4 for LIM5, LIM7, LIM13; n=6 for RGT Planet background). As the MGPA of LIM13 was intermediate of LIM5 and LIM7, the reduction in TGW and yield of LIM13 could not readily be attributed to the difference in MGPA (**Table 2**).

**Table 2:** Results of the field trial for mature grain phytase activity, thousand grain weight and plot yield.

	MGPA (FTU/Kg)	Index	TGW (grams)	Index	Plot yield (Kg)	Index
RGT Planet	840 ± 66	100	57.27 ± 2.35	100	6.78 ± 0.59	100
LIM5	1126 ± 119	134	55.22 ± 0.80	96	6.16 ± 0.25	91
LIM7	92 ± 37	11	56.51 ± 1.11	99	6.34 ± 0.32	94
LIM13	654 ± 53	78	48.09 ± 1.47 *	84	5.04 ± 0.24*	74

± indicates standard deviation. * indicate significant difference to RGT Planet (Tukey multiple comparison ANOVA, n=4 for LIM5, LIM7, LIM13; n=6 for RGT Planet).

### 3.3 Phytase and inositol phosphate dynamics during germination

**Figure 2:**
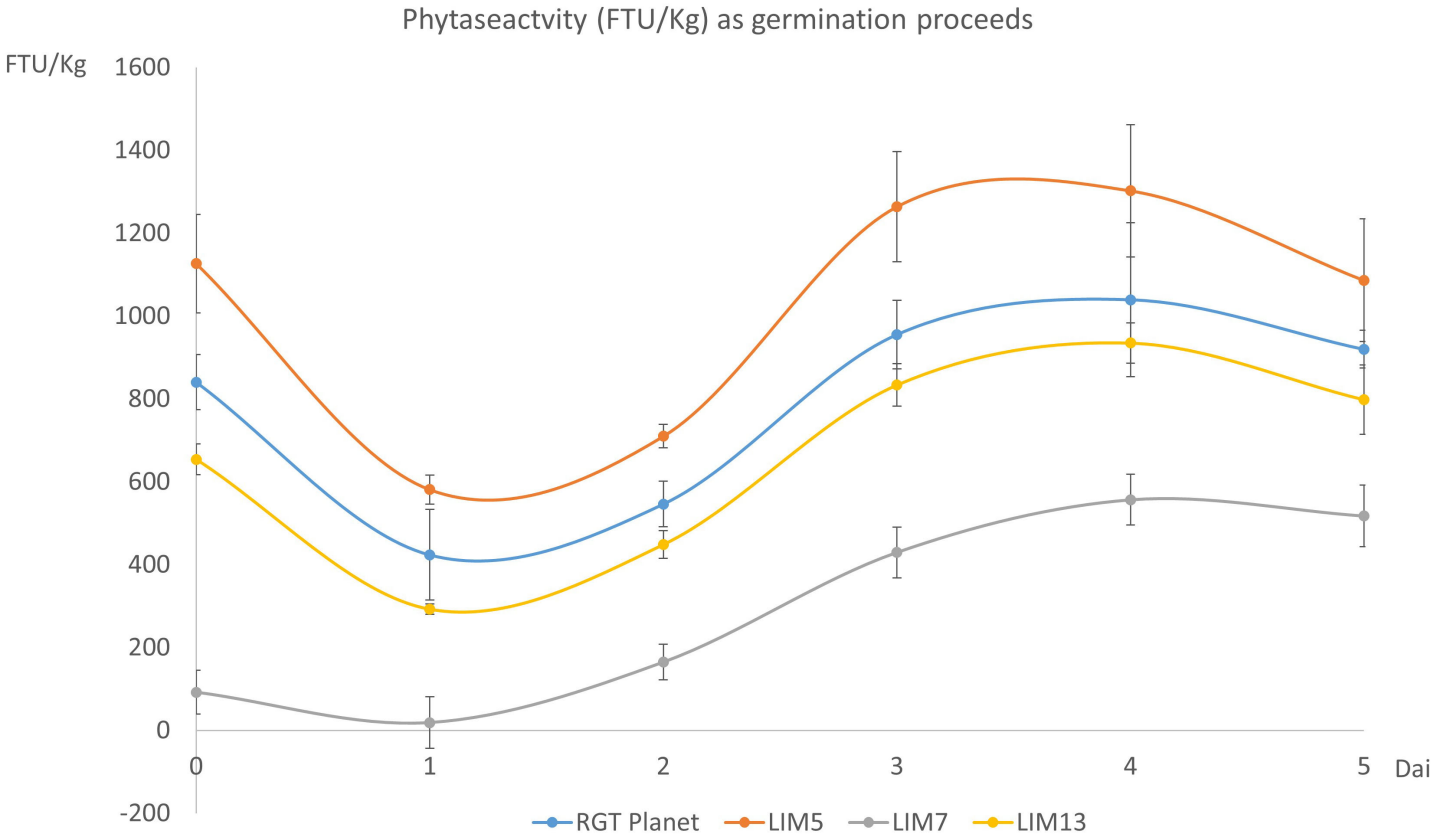
Phytase activity (FTU/Kg) in the mature grains (t=0) and during germination (day 1 to 5). Error bars show the standard deviation of four biological replicates. The difference in phytase activity was significant for all pairs of genotypes (n=4, p<0.05).

The phytase activity during germination followed a similar trend for the four lines. The activity initially decreased but recovered from day two onwards to reach a new maximum at day four. The initial ranking of the lines remained the same throughout the experiment but the lines with lower initial activities had a comparatively higher increase. Thus, the peak value was 24%, 16%, 604% and 43% higher than the initial value for RGT Planet, LIM5, LIM7 and LIM13, respectively (**Figure 2**).

**Figure 3:**
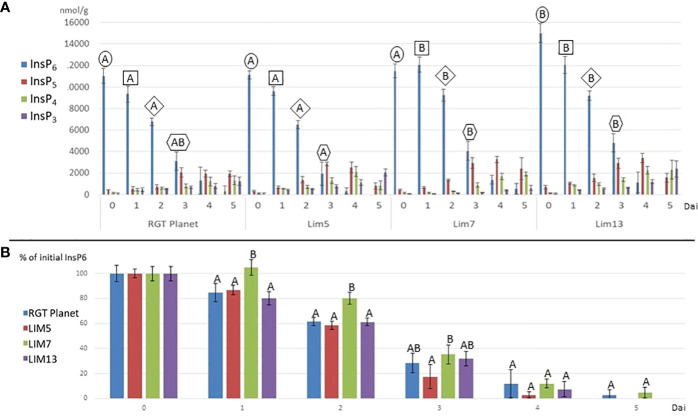
**(A)** Inositol phosphates (InsP_3_-InsP_6_) during germination (days after imbibition (dai) 1 to 5) and in the mature grains, i.e. MGPA (dai = 0) for the parent RGT Planet and mutants LIM5, LIM7 and LIM13. Letters above the columns denote if the InsP6 concentration is different (different letters) or not (same letters) from other concentrations from the same timepoint (n=4, p<0.05). The frame of the letters indicate samples from the same timepoint. E.g. all samples from 1 dai are framed by a square. No InsP6 value was different from the others at day four and five (not shown). **(B)** InsP_6_ as percent of the initial content at days after imbibition 0 to 5. Letters above the columns denote if the InsP6 concentration is different (different letters) or not (same letters) from other concentrations from the same timepoint (n=4, p<0.05).

The hydrolysis of phytate to lower inositol phosphates proceeded at a pace generally reflecting the phytase activity (**Figure 3**). LIM7 had no detectable hydrolysis for the first day and remained behind RGT Planet until day three. The phytate hydrolysis in LIM5 was not different from RGT Planet and LIM13. It was faster than LIM7 until day four when little InsP_6_ remained in all lines. LIM5 was the only line, which differed from LIM7 at day three (**Figure 3B**). LIM13 had a notably higher initial phytate content making it difficult to draw a direct comparison to the parent and sister mutants. Indexing the phytate values after the initial concentration in the respective mutants and the parent revealed that phytate hydrolysis in LIM13 proceed at the same pace as in parent line RGT Planet (**Figure 3B**). The lower inositol phosphates appeared in overlapping waves. InsP_5_ peaked at day three or four for all lines. The concentration of InsP_4_ increased with time in the parent and all mutants except LIM5 where the concentration decreased from day 4 to 5. InsP_3_ increased throughout the experiment for all lines but at a notably slower rate in LIM7 (**Figure 3A**).

### 3.4 *PAPhy_a* and *PAPhy_b* expression in mutants and parent

**Figure 4:**
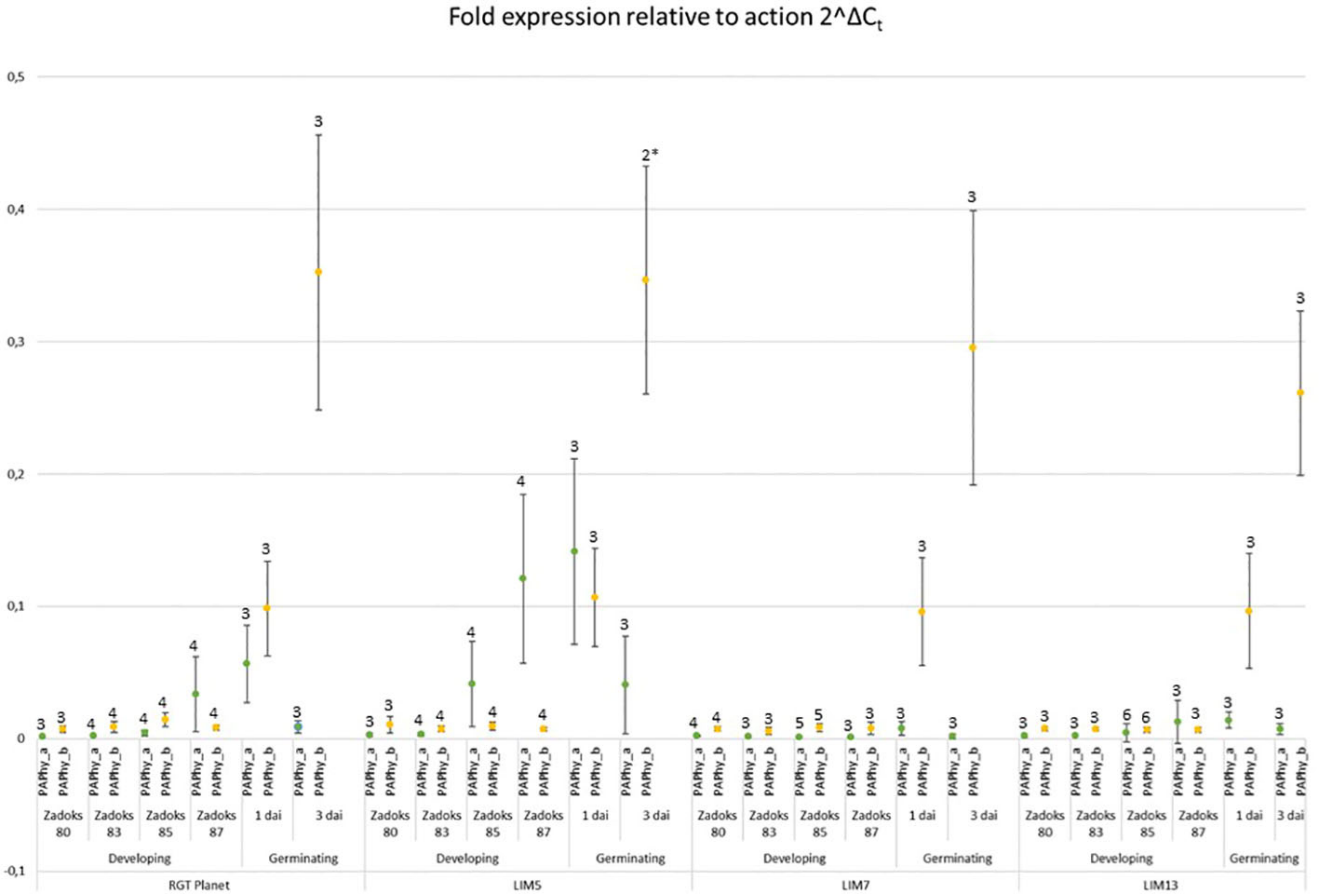
Expression of *PAPhy_a* (green data points) and *PAPhy_b* (yellow data points) relative to actin in the parent RGT Planet and LIM mutants during development and germination. Error bars represent the standard deviation of all replicates and the number above the error bars show the number of biological replicates (each measured in technical triplicate). One biological repetition was discarded as an outlier (Grubbs test; p<0,05 and high variation of technical replicates). The data point from the remaining two biological repetitions is marked with an * and should be interpreted with caution. It is, however in good agreement with the corresponding data points from the other lines.

The expression of both phytases was at least a hundred-fold lower than actin at Zadoks stage 80 and 83, the early phases of grain filling (**Figure 4**). This continued through Zadoks 85 except for LIM5, in which *PAPhy_a* was induced at this point. Expression increased further at Zadoks 87, at which point both the parent RGT Planet and, to a much lesser extent, LIM13 express *PAPhy_a*. Expression *of PAPhy_b* remained negligible in all lines throughout grain filling. LIM7 did not express either phytase at high levels during grain filling. Both phytases were expressed in germinating seeds of RGT Planet and LIM5 one day after imbibition. LIM7 and LIM13 expressed only negligible amounts of *PAPhy_a* during germination. All lines expressed *PAPhy_b* during germination and the expression level increased at least three-fold from day one to day three. The variation in *PAPhy_b* between biological replicates was noticeable three days after imbibition. This is not surprising, since each biological replicate comprised of just two seeds sampled at a time when expression is rapidly increasing. Nevertheless, the average *PAPhy_b* expression level at a given sampling time was consistent between lines.

## 4 Discussion

The ability to modify the location, timing and strength of the expression of specific plant genes has great potential in plant breeding. This field was previously limited by the difficulties encountered when screening mutant populations for subtle phenotypes, e.g. in biochemical composition. DNA based screening methods such as TILLING (McCallum et al., 2000) and recently FIND-IT (Knudsen et al., 2022) has made it more feasible to discover regulatory mutants based on DNA sequence analysis. Conserved CREs are clues to promoter regions where the expression level is likely to be affected by a mutation. The same CREs may be binding sites for enhancers or repressors depending on the context and timing (Schmitz et al., 2021). Thus, the direction of expression changes can only be predicted *a priori* in very well characterized promoters. Nevertheless, mutations with significant effect on the expression level -whether up or down- are valuable contributions to the study of promoter function and its roles in agronomic traits. Here we used FIND-IT to identify promoter mutants in a mildly mutagenized but large barley population. This approach is genotype independent and thus allowed us to use a contemporary elite barley variety, RGT Planet (Knudsen et al., 2022).

Single base mutations in the desired region, significantly affecting *PAPhy_a* gene expression were identified. It was possible to select homozygous mutants without any obvious phenotype and two mutant lines - LIM5 and LIM7 - retained their agronomical elite variety performance. The third mutant line - LIM13 - showed notably poorer performance with reductions in the yield parameters of 26 and 16% respectively. There was no correlation between the MGPA trait and the yield reductions, so they appear to be caused by unknown background mutations.

The mutation in LIM5 change the composite CRE to resemble that of other Triticeae tribe cereals, including wheat and rye. This Triticeae consensus sequence is an odd base palindrome with perfect symmetry extending five base pairs to either side of the central guanine (**Figure 1B**). The LIM5 mutant displayed higher *PAPhy_a* expression during grain filling, resulting in increased MGPA which reached the levels in wheat and rye (Eeckhout and De Paepe, 1994; Viveros et al., 2000). It should be noted that individual barley cultivars have been encountered, which outperform wheat cultivars in the greenhouse environment (Madsen et al., 2013). It was not investigated whether this reflects the variation within wheat and barley cultivars or whether it was caused by a higher response to environmental conditions in barley. To our knowledge, LIM5 is the only reported barley *PAPhy_a* mutant with increased MGPA. This include CRISPR-Cas and TALEN mutants reported by Holme et al. (2017b) all of which showed reduced MGPA. This may be related to fact that all the site directed nuclease generated plants possessed deletions, insertions or a combination of both whereas the current chemically mutagenized plants possess predominantly single base substitutions. More examples are needed to confirm or dismiss this possible connection between the type of mutation and the likelihood of the gene expression being increased or decreased, respectively.

In LIM13, the central nucleotide of the odd base palindrome was changed (**Figure 1B**). Combined with the preexisting deviation from the Triticeae consensus this provides a notable disruption of oddbase palindrome/GCN4 motif. A strong reduction of *PAPhy_a* expression was observed but ultimately this only reduced MGPA by 22%. One possible explanation is that the reduction of expression is partially buffered at the posttranscriptional level. It is possible that *PAPhy_a* mRNA translation is influenced by RNA methylation or microRNA interference (Vasudevan, 2012; Yue et al., 2019). Alternatively, the mutant may express significant amounts of phytase in the very last stages of ripening, not sampled here. Certainly, RGT Planet as well as LIM5 also increase expression until the last data point. On the other hand, RGT Planet and LIM5 have high amounts of transcript on the first day of germination, whereas LIM13 do not. Thus, expression and translation of *PAPhy_a* in late seed maturation and the source of early germination transcripts (stored vs *de novo* synthesized) warrant further investigation. The rate of phytate hydrolysis in LIM13 was similar to that of the parent cultivar RGT Planet (**Figure 3B**). This may be related to the higher phytate concentration in LIM13 since higher substrate concentrations generally increase the rate of enzymatic reactions. The mutations in LIM5 and LIM13 affect the GCN4 part of the composite CRE. It is known that this element integrates expression of the barley storage protein C-hordein with nitrogen availability (Müller and Knudsen, 1993). It remains to be investigated if *PAPhy_a* expression is also regulated by nitrogen availability and whether or not the mutations presented here has an effect on such a mechanism, if present. Furthermore, it would be worth investigating if barley storage protein expression under different nitrogen regimes could be optimized by induced mutations in GCN4 motif.

LIM7 is mutated at a position between the composite CRE and the first TATA-box which has not previously been assigned as a CRE by *in silico* analysis (Madsen et al., 2013). On manual reexamination, we identified high similarity to the Dof transcription factor core recognition sequence ((T/A)AAAG) adjacent to the mutation (**Figure 1A**). In antisense, the sequence has particularly high similarity to a Dof recognition sequence variant, the pyrimidine box (CCTTT) that has been implicated in Dof mediated response to gibberellin in the barley aleurone layer (Isabel-LaMoneda et al., 2003). The three bases preceding the adenine triplet are often taken to be part of the binding motif e.g. in the prolamin box motif (also known as the endosperm box) (Müller and Knudsen, 1993), but there is also evidence that they are not essential ( (Yanagisawa and Schmidt, 1999). Another possibility is that the Dof recognition sequence is part of a bifactoral CRE where the other element is still unknown. This would be a parallel to the well described bifactoral element of the GCN4 motif (recognition sequence of SPA, a bZIP) and the prolamin box in cereal storage protein promoters (Albani et al., 1997; Mena et al., 1998). The adjacent sequence has some sequence symmetry, which supports this hypothesis (CCACAAGGCACC; the central AAGG mirrors CCAC). The symmetric sequence, except for the first C, as well as the putative Dof recognition sequence are highly conserved in the diverse set of Triticeae *PAPhy_a* sequences we reported previously (Madsen et al., 2017). The only exception is the locus on the B genome of tetra- and hexaploid wheat where the elements are entirely missing. Currently, is not known whether these loci are transcribed. There are homeologs on the A and D genomes, rendering the B genome copy redundant.

The dynamics of phytase induction and relationship to inositol phosphate profiles of germinating seedlings has been little studied in cereals. One exception is the study of (Centeno et al., 2001), which contrary to our study did not observe a decrease in phytase activity after one day of germination in barley. While we observed an approximate doubling of phytase activity between day 1 and day 4, the earlier study noted a progressive increase in phytase activity to three times the initial activity on day five. Phytase activities were always notably higher than the current study, but even so, one third of the initial phytate remained after five days of germination, which is considerably more than reported here. The same trends for germinative phytase activity were found by (Greiner et al., 2000), but with activity levels much closer to the current findings. Four barley cultivars were examined in parallel by (Bouajila et al., 2020). Three of the cultivars had MGPA in the same range, 600 – 800 FTU/Kg, whereas the fourth had twice that activity (1600 FTU/Kg). The four cultivars reached the same level of, 1000- 1200 FTU/Kg on the first day of germination so the cultivar with high MGPA had lost activity whereas the others had increased their activity. The phytase activity proceeded to increase to reach a maximum on day 8 with, 6000- 8000 FTU/Kg. The phytate content decreased throughout the 12-day experiment and more than half was left after five days. A notably different result was obtained by (Egli et al., 2002) who found a marked decrease in phytase activity upon soaking the seeds which failed to recover even after three days of germination. Thus, the general trend in the literature is that phytase activity increases for the first four to eight days of barley germination. An initial decrease of activity has been reported in some studies besides the current (Egli et al., 2002; Bouajila et al., 2020). We have not identified any obvious technical reasons why some observe the initial decrease while other do not, but we wish to point out that several processes with potential to influence the measured activity are ongoing simultaneously. 1) Imbibition activates the preformed phytase (PAPhy_a) which initiates hydrolysis of seed phytate as shown in **Figure 3**. This changes the seed-derived pool of inositol phosphates, which are, inevitably, an additional source of substrate on top of the exogenous sodium phytate supplied for the assay. 2) The internal hydrolysis leading up to the point of assay also produces free phosphate, the compound measured in the assay. This background phosphate was subtracted in the current study, but obviously different ways of handling the background may lead to very different assay results. 3) Biosynthetic pathways using phosphate or inositol phosphates are active at the same time as the hydrolytic pathway. This is in part because of the growth of shoot and roots initiated from the embryo but even the aleurone layer has phytate biosynthesis (Brearley and Hanke, 1996). Irrespective of these minor discrepancies between studies, the overall picture is clear. The phytate content invariably decrease as germination proceeds and the process takes about one week to complete.

In the current study, the concept of promoter modification using FIND-IT technology was demonstrated. New cis-regulatory alleles for higher and lower MGPA were identified. The selected CRE motifs are shared with a range of seed expressed genes like key cereal and legume storage genes, and the current results demonstrates a concept for modulating individual gene expression levels that may be used for genes with similar CRE composition. For *PAPhy_a*, the preformed phytase activity correlated with phytate hydrolysis and the appearance of lower inositol phosphates in the first days of germination. This confirms a role for *PAPhy_a* as a booster of phytate hydrolysis in the initial stage of germination.

## Data availability statement

The original contributions presented in the study are included in the article/supplementary files. Further inquiries can be directed to the corresponding author.

## Author contributions

CM: Conceptualization, Formal analysis, Investigation, Visualization, Writing – original draft. CB: Formal analysis, Investigation, Writing – review & editing. JH: Investigation, Methodology, Resources, Writing – review & editing. HB-P: Conceptualization, Funding acquisition, Project administration, Supervision, Writing – review & editing.
